# Controversias en Cardiología. Parte 1. ¿Debo tratar un síndrome coronario crónico de alto riesgo invasivamente desde el inicio? Sí, en la mayoría de casos

**DOI:** 10.47487/apcyccv.v1i4.86

**Published:** 2020-12-31

**Authors:** César Antonio Ortiz Zegarra, Piero Custodio Sánchez, Paol Rojas de la Cuba, Gorki E Mori Pinedo, Ricardo Coloma Araniya, Bertha Aidee Gonzales Álvarez, Christian Nolte Rickards

**Affiliations:** 1 Médico asistente del Servicio de Cardiología Intervencionista. Instituto Nacional Cardiovascular - INCOR. Lima. Perú. Servicio de Cardiología Intervencionista Instituto Nacional Cardiovascular - INCOR Lima Perú; 2 Médico asistente del Servicio de Cardiología Intervencionista. Hospital Nacional Almanzor Aguinaga Asenjo. Chiclayo, Perú. Servicio de Cardiología Intervencionista Hospital Nacional Almanzor Aguinaga Asenjo Chiclayo Perú; 3 Médico asistente del Servicio de Cardiología Intervencionista. Hospital Nacional Guillermo Almenara. Lima, Perú. Servicio de Cardiología Intervencionista Hospital Nacional Guillermo Almenara Lima Perú; 4 Coordinador CDTE Clínica San Felipe. Lima, Perú. CDTE Clínica San Felipe Lima Perú; 5 Jefe de la Unidad de Hemodinámica del Hospital Central FAP. Lima, Perú. Unidad de Hemodinámica del Hospital Central FAP Lima Perú; 6 Cardióloga de la Clínica Tezza. Lima, Perú. Clínica Tezza Lima Perú; 7 Médico asistente del servicio de cardiología intervencionista. Instituto Nacional Cardiovascular - INCOR. Lima, Perú. servicio de cardiología intervencionista Instituto Nacional Cardiovascular - INCOR Lima Perú; a Secretario de ética de la Sociedad Peruana de Hemodinámica e Intervencionismo Endovascular (SOPHIE). Sociedad Peruana de Hemodinámica e Intervencionismo Endovascular (SOPHIE); b Secretario de filiales de Sociedad Peruana de Hemodinámica e Intervencionismo Endovascular (SOPHIE). Sociedad Peruana de Hemodinámica e Intervencionismo Endovascular (SOPHIE); c Secretario de la Sociedad Peruana de Hemodinámica e Intervencionismo Endovascular (SOPHIE). Sociedad Peruana de Hemodinámica e Intervencionismo Endovascular (SOPHIE); d Vicepresidente de la Sociedad Peruana de Hemodinámica e Intervencionismo Endovascular (SOPHIE). Sociedad Peruana de Hemodinámica e Intervencionismo Endovascular (SOPHIE); e Expresidente de la Sociedad Peruana de Hemodinámica e Intervencionismo Endovascular (SOPHIE). Sociedad Peruana de Hemodinámica e Intervencionismo Endovascular (SOPHIE); f Secretaria de Economía de la Sociedad Peruana de Hemodinámica e Intervencionismo Endovascular (SOPHIE). Sociedad Peruana de Hemodinámica e Intervencionismo Endovascular (SOPHIE); g Presidente de la Sociedad Peruana de Hemodinámica e Intervencionismo Endovascular (SOPHIE). Sociedad Peruana de Hemodinámica e Intervencionismo Endovascular (SOPHIE)

**Keywords:** Enfermedad Coronaria, Angina de Pecho, Revascularización Miocárdica, Coronary Disease, Angina Pectoris, Myocardial Revascularization

## Abstract

El síndrome coronario crónico (SCC) previamente conocido como enfermedad coronaria estable, es la principal causa de mortalidad en el mundo; en el Perú es una de las más importantes. Esta patología presenta una naturaleza dinámica que resulta en diferentes escenarios clínicos que pueden ser modificados mediante diversas opciones terapéuticas, una de ellas es el tratamiento intervencionista coronario, principalmente en pacientes de alto riesgo isquémico definido como una isquemia mayor al 10% de toda la masa ventricular izquierda. Por esta razón analizamos la información más relevante y actual disponible para concluir que el tratamiento del síndrome coronario crónico de alto riesgo isquémico, luego de una evaluación individual, correspondería a un manejo invasivo desde el inicio que, si bien no impactarían en la mortalidad o eventos cardiovasculares, sí contribuiría a mejorar la calidad de vida, considerando, además, la incompleta disponibilidad de todas las opciones terapéuticas para el manejo sintomático de esta patología, el limitado acceso al manejo de eventos cardiovasculares agudos en nuestro medio así como el riesgo de efectos adversos e interacciones medicamentosas.

## Abstract (en)

Chronic coronary syndrome (SCC) previously known as stable coronary disease, is the main cause of mortality in the world, and it is one of the most important in Peru. This pathology has a dynamic nature that results in different clinical scenarios that can be modified through various therapeutic options, one of which is coronary interventional treatment, mainly in patients with high ischemic risk defined as ischemia greater than 10% of the entire left ventricular mass. For this reason, we have analyzed the most relevant and current information available, concluding that the treatment of high ischemic risk´s chronic coronary syndrome, after an individual evaluation, would correspond to an invasive management from the beginning, although it would not impact on mortality or cardiovascular events, it would contribute to improve quality of life; also we should consider the incomplete availability of all the therapeutic options for the symptomatic management of this disease, the limited access to the management of acute cardiovascular events in our country, as well as the risk of adverse effects and drug interactions.

La enfermedad coronaria estable, actualmente conocida como síndrome coronario crónico (SCC)^1^ forma parte de un abanico de enfermedades cardiovasculares que a nivel mundial son la principal causa de mortalidad^2^; en el Perú la tasa de mortalidad por esta causa es aproximadamente del 28,7%^3^. El SCC se caracteriza por acumulación de placa aterosclerótica en arterias epicárdicas; presenta una naturaleza dinámica que resulta en diferentes escenarios clínicos, este proceso puede ser modificado aplicando desde intervenciones en el estilo de vida y medidas farmacológicas, hasta el intervencionismo coronario^1^, por esta razón es importante analizar las pruebas complementarias y las opciones terapéuticas basadas en evidencia científica actual para optimizar el manejo en los pacientes basados en el contexto de nuestra realidad nacional con un sistema de salud poco eficiente. El objetivo de la presente revisión es analizar la información actual disponible que nos permita orientar y analizar el manejo intervencionista en este grupo de pacientes.

## Estudios observacionales (nivel de evidencia B)

Un estudio realizado por Hachamovitch R *et al.*^4^, utilizando la tomografía computarizada por emisión de fotón único (SPECT) cardiaco, en más de diez mil pacientes con SCC sin infarto agudo de miocardio previo, mostró una relación directa entre el porcentaje de isquemia y el riesgo de revascularización, y una tendencia a mejor supervivencia en quienes tenían un tratamiento invasivo dentro de los 60 días del diagnóstico, sobre todo en presencia de cargas isquémicas elevadas, definidas como 20% o más del total de masa ventricular izquierda (que en el grupo de revascularizados fue casi 40%, y en el grupo de solo tratamiento médico fue 2,5%, aproximadamente).

En un estudio retrospectivo realizado por Yoda *et al.*^5^, se encontró en 3581 pacientes con angina estable estratificada con SPECT cardiaco, una incidencia significativamente menor de eventos cardiacos mayores en pacientes con isquemia > 10% que fueron revascularizados tempranamente dentro de los 60 días de la prueba, versus aquellos que no fueron revascularizados (7% versus 16,8%, p= 0,0036). Además, Patel *et al.*^6^, encontraron en una cohorte contemporánea de 16 029 pacientes con diagnóstico o sospecha de enfermedad arterial coronaria, que fueron sometidos a tomografía por emisión de positrones (PET), que pacientes con mayor isquemia tuvieron una mejora de la sobrevida con la revascularización temprana (dentro de los 90 días posteriores al examen), con un potencial punto de corte de isquemia de 5%.

En otro estudio, Miller *et al*. ^7^, encontraron en un registro observacional unicéntrico de 54 522 pacientes con enfermedad arterial coronaria estable y SPECT, que tanto la intervención coronaria percutánea y la cirugía de revascularización miocárdica, en presencia de isquemia moderada a severa, fueron asociadas con una reducción de mortalidad por todas las causas.

**Figura 1:**
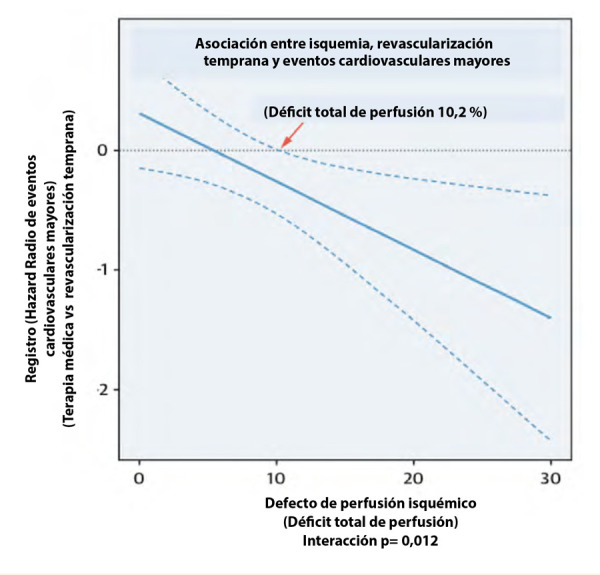
Revascularización temprana, eventos cardiovasculares mayores e isquemia miocárdica ^9^^).^

Azadani *et al*., en un registro internacional multicéntrico prospectivo de 19 088 pacientes con enfermedad coronaria estable y SPECT, con un seguimiento medio de 4,7 +/- 1,6 años, encontraron una reducción significativa de los eventos cardiovasculares mayores en pacientes con isquemia > 10,2% cuantificada automáticamente, que fueron revascularizados tempranamente ^8^**(**Figura 1**)**.

De la misma manera, un reporte reciente de Parikh *et al.*, en pacientes con enfermedad coronaria isquémica estable y estenosis intermedias angiográficamente (40 - 69% por estimación visual), encontraron que el uso de la reserva fraccional de flujo fue asociado a una mortalidad significativamente menor a 1 año ^9^.

Recientemente, en el registro multicéntrico CLARIFY ^10^ que incluyó 45 países de diversas partes del mundo, con algunos de Latinoamérica (2227 pacientes provenientes de Brasil, Argentina y México) entre noviembre de 2009 y junio de 2010, con un tiempo de seguimiento de 5 años en promedio, presentó datos importantes sobre el pronóstico de estos pacientes en países latinoamericanos, mostrando mayores tasas de muerte cardiovascular e infarto agudo de miocardio no fatal con respecto a otras regiones del mundo (HR=1,61; 95% CI: 1,38-1,88 análisis multivariado)*.* Lo que es importante considerar al momento de aplicar guías internacionales de realidades distintas a la nuestra.

## Estudios aleatorizados (nivel de evidencia A)

A pesar que en el estudio COURAGE ^11^, publicado en el 2007, que incluyó 2287 pacientes con angina estable provenientes de EE.UU. y Canadá, con un seguimiento de casi cinco años, mostró que el tratamiento invasivo no disminuyó la mortalidad (alrededor de 8% en ambos grupos), ni el compuesto de muerte cardiaca, infarto agudo de miocardio y eventos cerebrovasculares, en comparación con el tratamiento médico óptimo (TMO) (20,0% vs. 19,5%;p=0,62); es importante resaltar que fueron excluidos pacientes de alto riesgo como pacientes con fracción de eyección menor a 30%; lesión severa de tronco coronario izquierdo, y angina de grado avanzado o revascularización previa los últimos seis meses. Además, el grupo de TMO tuvo una mayor necesidad de revascularización frente al grupo invasivo (32,6% vs 21,1% HR, 0,60; 95% CI, 0,51-0,71; p<0,001) en los primeros meses de seguimiento, aunque a los cinco años desapareció esta diferencia. También es importante considerar que la revascularización disminuyó la presencia de angina y la carga isquémica frente a quienes recibieron solo el tratamiento médico (78% frente al 52% de los pacientes con grado moderada a severo de isquemia). Otro punto importante es el desarrollo de *stents* medicados de nueva generación que han demostrado menores eventos cardiovasculares que los utilizados en este estudio, y podrían tener resultados más favorables para el grupo invasivo como lo refieren algunas guías internacionales^12^^-^^14^ o como lo muestran algunos metaanálisis de síndrome coronario crónico^15^*.*

Estudios como el SCOT-HEART ^16^ donde el uso de la angiotomografía coronaria (angioTAC) en pacientes con SCC en tratamiento médico convencional, evidenció una reducción significativa de eventos cardiovasculares mayores como muerte e infarto agudo de miocardio no fatal (2,3% vs. 3,9% p= 0,004 [HR, 0,59; 95% CI, 0,41, 0,84]) a los cinco años de seguimiento luego del manejo invasivo del grupo en quienes se realizó la angioTAC. Sin embargo, se debe considerar que ante un ritmo cardiaco irregular y calcificación coronaria severa esta prueba puede tener poca precisión diagnóstica y sobreestimar el grado de severidad, además de los riesgos por el uso de radiación o contraste nefrotóxico que deben sopesarse respecto a los beneficios cuando se indiquen a pacientes jóvenes o con enfermedad renal importante.

El Estudio FAME 2 ^17^ mostró a los 5 años de seguimiento que en pacientes con SCC, el tratamiento invasivo con angiografía coronaria y revascularización guiada por reserva de flujo fraccional (FFR), con un punto de corte FFR ≤ 0,80, fue superior frente al tratamiento médico en el objetivo primario compuesto por muerte, infarto agudo de miocardio y revascularización de urgencia de 13,9% vs. 27,0% (HR 0,46; IC 95% 0,34, 0,63, p <0,001).

El estudio ORBITA^18^, publicado en el 2017, comparó en pacientes con angina estable la intervención coronaria percutánea (ICP) frente a la TMO, realizando un cateterismo cardiaco en ambos grupos sin conocimiento de los pacientes si se les había realizado angioplastia coronaria, y aleatorizó 1:1 la ICP contra placebo. Se encontró que la ICP no mejoraba el tiempo de ejercicio medido por prueba de esfuerzo, sin embargo, la muestra fue pequeña, menos de 105 y 95 pacientes respectivamente; se incluyó solo a pacientes con enfermedad coronaria uniarterial severa determinada por angiografía, en tanto que la severidad de las lesiones no fue validada por un laboratorio central. Además, el tiempo de seguimiento fue de solo seis semanas.

También es importante considerar el estudio ISCHEMIA ^19^ publicado el 2020 donde se evaluó si el intervencionismo coronario percutáneo de rutina (ICPr) asociada a TMO reducía eventos de muerte cardiovascular, infarto de miocardio y rehospitalizaciones por angina inestable, insuficiencia cardiaca o arresto cardiaco, comparada a la TMO sola, en pacientes con angina estable y evidencia de isquemia reversible moderada o severa. En un seguimiento de 3,2 años no hubo diferencias significativas entre ambos grupos (ICP + TMO [13,3%] vs. TMO [15,5%]), por lo que se concluyó que la estrategia invasiva inicial comparada a la TMO no reducía el riesgo de eventos cardiovasculares o muerte de cualquier causa. Cabe resaltar que este estudio trato de reducir el sesgo de selección de pacientes visto en estudios previos, conociendo la anatomía coronaria previa a la randomización mediante tomografía espiral multicorte (TEM) de arterias coronarias (excluyendo a pacientes con enfermedad de tronco coronario izquierdo (TCI) significativa y arterias coronarias sin lesiones significativas) además de realizar medidas meticulosas en la adherencia a la TMO (se excluyeron a pacientes insatisfechos con la TMO o que tenían antecedentes de incumplimiento a la medicación para minimizar el *crossover*).

En este estudio no se incluyó a pacientes con una filtración glomerular menor a 30 mL/min, evento coronario agudo reciente (menor a dos meses), estenosis ≥ 50% de TCI evaluado con TEM de arterias coronarias, pacientes con fracción de eyección del ventrículo izquierdo < 35%, insuficiencia cardiaca en clase funcional de la NYHA III - IV, angina inaceptable a pesar de terapia médica a dosis máximas aceptables, antecedente de ICP o CABG doce meses previos; justamente este tipo de pacientes donde la ICP tiene un mayor impacto en eventos cardiovasculares a largo plazo. 

Algunos puntos que resaltar en el estudio ISCHEMIA son: 1) La tasa de eventos cardiovasculares tempranos (infarto de miocardio [IM] asociado a ICP o tipo 4a) fueron mayores en el grupo de ICP mientras que los eventos cardiovasculares tardíos (infarto espontaneo o tipo1) fueron menores; estas diferencias entre ambos grupos fue por la mayor tasa de infarto periprocedimiento (IMp) en el grupo de ICP comparado al grupo de TMO (5,3% vs. 3,4%), esto según el uso de la definición de IMp, en el protocolo inicial usa como definición primaria de IMp a la elevación de CPKmb como prueba diagnóstica de infarto tipo 4A y no la troponina (porque los límites de decisión de infarto en el lugar de estudio pueden ser, o no, los mismos que los límites de los fabricantes); pero durante el estudio se utilizó como definición de IM tipo 4a; la que los autores denominaron como definición secundaria de IMp que fue la elevación de troponina sobre el percentil 99% independiente de los límites de referencia del fabricante, esto asociado a criterios electrocardiográficos y angiográficos; cabe resaltar que estudios previos muestran que los infartos espontáneos (IM tipo1) confieren un mayor riesgo de muerte que los IM tipo 4A. 2) Algunas limitaciones descritas en el estudio son: a) La reducción del poder estadístico para reducir el tamaño muestral del estudio (muestra inicial programada de 8000 se redujo a 5179 pacientes) ^(^^20^. b) reducción del periodo de estudio a 3,2 años (protocolo inicial a cuatro años) que según menciona Braunwald «Es posible que el ISCHEMIA haya terminado antes de una sustancial diferencia a favor de la estrategia invasiva». c) el laboratorio central del estudio no confirmó el grado de isquemia en el 14% de pacientes que fueron randomizados, mientras que el 12% tuvo prueba de isquemia de bajo riesgo; además, en un tercio de pacientes se incluyó a la prueba de esfuerzo como prueba de isquemia inducible previo, a pesar de su baja sensibilidad. 3) No se realizaron pruebas de isquemia invasivas tipo FFR o *Instantaneous Wave-Free Ratio* (iFR) en la mayoría de casos y tampoco se menciona que valor de corte utilizaron para decidir la revascularización de ese vaso ^21^. Hubo una modesta mejoría sintomática en el grupo de ICP, especialmente en pacientes con angina diaria/semanal (50% de pacientes estuvo libre de angina después de un año en el grupo de ICP y solo 20% en el grupo de TMO), cabe indicar que el 34% de pacientes no tenían angina al inicio del estudio ^22^. 4) Hasta el 28% del grupo de TMO sola, fue sometido a tratamiento invasivo, y unas de las causas más importantes fue no adherencia o falla al tratamiento (8,1 y 3,9%, respectivamente). 5) Dentro de Latinoamérica participaron Argentina y Brasil con 425 pacientes; Perú participo en el estudio de valoración de calidad de vida (cuatro pacientes llenaron el cuestionario), no se tuvo pacientes incluidos en el estudio original. Con tan poca cantidad de pacientes de Latinoamérica es controversial la extrapolación de estos resultados a nuestra comunidad sabiendo de las diferencias existentes entre regiones. Asimismo, se realizó un estudio en un centro hospitalario de tercer nivel para ver la implicancia clínica de este estudio y se evidenció que un porcentaje pequeño (28,4%) con síndrome coronario crónico cumple los criterios de inclusión y exclusión de este estudio. Este resultado indicaría que el impacto de este estudio en la práctica habitual sería limitado ^23^.

En conclusión, la terapia invasiva de rutina en SCC puede no ser necesaria para prevenir eventos adversos en pacientes oligosintomáticos, mientras que la ICP precoz es segura para pacientes que prefieren reducir la cantidad de medicación, los poco tolerantes a la medicación, o aquellos que tienen síntomas persistentes a pesar de la medicación; además, siempre hay que analizar de manera individualizada la probabilidad que la revascularización coronaria óptima pueda ser alcanzada con escasas complicaciones por el procedimiento, ya que estos procedimientos son más seguros en centros hospitalarios donde existe una cantidad de procedimientos anuales aceptables.

Recientemente se ha publicado un subestudio del ISCHEMIA^24^ en él se incluyen pacientes con historia de falla cardiaca o fracción de eyección moderadamente disminuida entre 35 y 45%, se muestra que quienes recibieron manejo invasivo inicial tuvieron menos eventos cardiovasculares como muerte cardiaca, infarto agudo de miocardio no fatal, hospitalización por angina inestable o falla cardiaca, o paro cardiaco resucitado, durante un seguimiento de cuatro años (17,2% vs. 29,3%; 95% IC: -22,6, -1,6%, p<0,05) por lo que sería importante considerar a este grupo de paciente para manejo invasivo inicial, aunque se requieren más estudios al respecto.

## Metaanálisis de ensayos clínicos (nivel de evidencia A)

**Figura 2:**
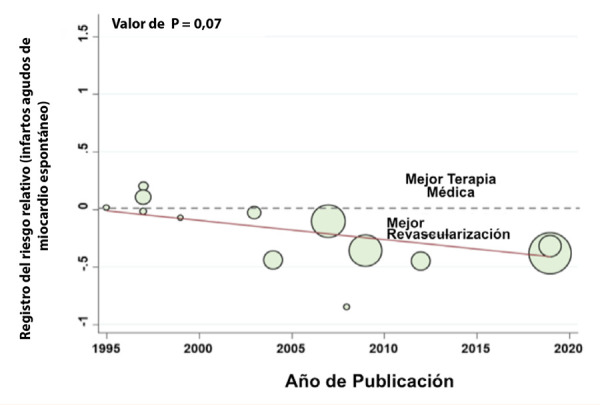
Disminución de infartos agudos de miocardio con terapia de revascularización precoz ^21^.

Finalmente, en el 2020 fue publicado un metaanálisis de catorce estudios clínicos aleatorizado, que incluyó 14 877 pacientes con síndrome coronario crónico y angina grado I o II de la Canadian Cardiovascular Society (CCS), fracción de eyección conservada excluyendo pacientes con enfermedad de tronco coronario izquierdo. Este análisis, si bien no mostró disminución en mortalidad con el tratamiento invasivo frente al manejo médico óptimo, encontró mejora en la presencia de angina (RR 1,10; 95% CI 1,05 - 1,15) sobre todo con el uso de *stents* medicados, que disminuyó los cuadros de angina inestable (RR 0,45, 95% CI 0,29-0,71; p = 0,003) y de infarto agudo de miocardio no asociados al procedimiento (RR 0,76, 95% CI 0,67, 0,85; p= 0,04) (Figura 2) con un tiempo de seguimiento de cuatro años y medio^25^.

## Conclusiones

El tratamiento de los pacientes con SCC debe plantearse de forma individualizada y considerando todos los factores asociados, haciendo uso de las mejores herramientas disponibles para la predicción de riesgos y adecuándose al entorno sanitario del paciente. Debemos considerar:

1. En pacientes de alto riesgo isquémico con carga isquémica entre 10 a 20%, la evaluación individual podría determinar un manejo invasivo inicial que, si bien no impactarían en la mortalidad o eventos cardiovasculares, sí contribuiría a mejorar su calidad de vida, considerando, además, la incompleta disponibilidad de todas las opciones terapéuticas para el manejo sintomático de esta patología, así como el riesgo de efectos adversos e interacciones medicamentosas que pueden llevar a falla de la terapia que se puede presentar en un porcentaje importante (12% en el grupo de solo medicación del estudio ISCHEMIA)^21^^)^ y requerir tratamiento invasivo.

2. En pacientes de muy alto riesgo isquémico como carga de isquemia mayor o igual a 20% (casi un 40% de los revascularizados en el estudio de Hachamovitch R, *et al.*) o con enfermedad severa de tronco (hasta un 8% de angiografías coronarias)^(26, 27)^ o fracción de eyección severamente disminuida (55% en el subestudio de ISCHEMIA tenían fracción de eyección moderadamente disminuida, y quienes recibieron revascularización tuvieron menos eventos cardiovasculares frente a los que solo recibieron tratamiento médico óptimo)^24^, deberíamos considerar el tratamiento invasivo lo antes posible debido al riesgo elevado de complicaciones cardiovasculares pues, si bien no existe mucha evidencia al respecto, la mayoría de estudios no incluyen a este tipo de pacientes en sus protocolos y en países con sistema de salud como el nuestro la atención de urgencia es muy limitada^28^.

3. En todos los casos de SCC debería darse una adecuada información al paciente sobre las opciones terapéuticas considerando riesgo-beneficio, la situación sociocultural y demográficas, además de los problemas asociados que puedan prever el fracaso de un adecuado tratamiento médico, siendo una mejor alternativa el manejo invasivo.

## References

[1] Knuuti J, Wijns W, Saraste A, Capodanno D, Barbato E, Funck-Brentano C (2020). 2019 ESC Guidelines for the diagnosis and management of chronic coronary syndromes The Task Force for the diagnosis and management of chronic coronary syndromes of the European Society of Cardiology (ESC). Eur Heart J.

[2] World Health Organization (2020). Disease burden and mortality estimates.

[3] Ministerio de Salud del Perú [Internet] (2020). Ministerio de Salud.

[4] Hachamovitch R, Hayes SW, Friedman JD, Cohen I, Berman DS (2003). Comparison of the Short-Term Survival Benefit Associated with Revascularization Compared with Medical Therapy in Patients with No Prior Coronary Artery Disease Undergoing Stress Myocardial Perfusion Single Photon Emission Computed Tomography. Circulation.

[5] Yoda S, Hori Y, Hayase M, Mineki T, Hatta T, Suzuki Y (2018). Correlation between early revascularization and major cardiac events demonstrated by ischemic myocardium in Japanese patients with stable coronary artery disease. J Cardiol.

[6] Patel KK, Spertus JA, Chan PS, Sperry BW, Thompson RC, Badarin FA (2019). Extent of Myocardial Ischemia on Positron Emission Tomography and Survival Benefit with Early Revascularization. J Am Coll Cardiol.

[7] Miller R, Bonow RO, Gransar H, Park R, Slomka PJ, Friedman JD (2020). Percutaneous or surgical revascularization is associated with survival benefit in stable coronary artery disease. Eur Heart J.

[8] Azadani P, Miller RJH, Sharir T, Diniz MA, Hu LH, Otaki Y (2021). Impact of Early Revascularization on Major Adverse Cardiovascular Events in Relation to Automatically Quantified Ischemia. JACC Cardiovasc Imaging.

[9] Parikh RV, Liu G, Plomondon ME, Sehested TSG, Hlatky MA, Waldo SW (2020). Utilization and Outcomes of Measuring Fractional Flow Reserve in Patients with Stable Ischemic Heart Disease. J Am Coll Cardiol.

[10] Sorbets E, Fox KM, Elbez Y, Danchin N, Dorian P, Ferrari R (2020). Long-term outcomes of chronic coronary syndrome worldwide insights from the international CLARIFY registry. Eur Heart J.

[11] Boden WE, O'Rourke RA, Teo KK, Hartigan PM, Maron DJ, Kostuk WJ (2007). Optimal Medical Therapy with or without PCI for Stable Coronary Disease. N Engl J Med.

[12] Neumann FJ, Sousa-Uva M, Ahlsson A, Alfonso F, Banning AP, Benedetto U (2019). Eur Heart J.

[13] Patel MR, Calhoon JH, Dehmer GJ, Grantham JA, Maddox TM, Maron DJ (2017). ACC/AATS/AHA/ASE/ASNC/SCAI/SCCT/STS 2017 Appropriate Use Criteria for Coronary Revascularization in Patients With Stable Ischemic Heart Disease A Report of the American College of Cardiology Appropriate Use Criteria Task Force, American Association for Thoracic Surgery, American Heart Association, American Society of Echocardiography, American Society of Nuclear Cardiology, Society for Cardiovascular Angiography and Interventions, Society of Cardiovascular Computed Tomography, and Society of Thoracic Surgeons. J Am Coll Cardiol.

[14] Cesar LA, Ferreira JF, Armaganijan D, Gowdak LH, Mansur AP, Bodanese LC (2014). Guideline for Stable Coronary Artery Disease. Arq Bras Cardiol.

[15] Windecker S, Stortecky S, Stefanini GG, da Costa BR, Rutjes AW, Di Nisio M (2014). Revascularization versus medical treatment in patients with stable coronary artery disease network meta-analysis. BMJ.

[16] (2015). SCOT-HEART investigators CT coronary angiography in patients with suspected angina due to coronary heart disease (SCOT-HEART): an open-label, parallel-group, multicentre trial. Lancet.

[17] Xaplanteris P, Fournier S, Pijls NHJ, Faeron WF, Barbato E, Tonino PAL (2018). Five-Year Outcomes with PCI Guided by Fractional Flow Reserve. N Engl J Med.

[18] Al-Lamee R, Thompson D, Dehbi HM, Sem S, Tang K, Davies J (2018). Percutaneous coronary intervention in stable angina (ORBITA) a double-blind, randomized controlled trial. Lancet.

[19] Maron DJ, Hochman JS, Reynolds HR, Bangalore S, O'Brien SM, Boden WE (2020). Initial Invasive or Conservative Strategy for Stable Coronary Disease. N Engl J Med.

[20] Spilias N, Zorach B, Denby K, Ellis S (2020). The role of ISCHEMIA in stable ischemic heart disease. Cleve Clin J Med.

[21] Dahal S, Budoff MJ (2020). Failed ISCHEMIA trial or failed ischemia testing. J Invasive Cardiol.

[22] Spertus JA, Jones PG, Maron DJ, O'Brien SM, Reynolds HR, Rosenberg Y (2020). Health-Status Outcomes with Invasive or Conservative Care in Coronary Disease. N Engl J Med.

[23] Meier D, Mahendiran T, Fournier S (2020). Will ISCHEMIA change our daily practice. Cardiovasc Diagn Ther.

[24] Lopes RD, Alexander KP, Stevens SR, Reynolds HR, Stone GW, Piña IL (2020). Initial Invasive versus Conservative Management of Stable Ischemic Heart Disease Patients with a History of Heart Failure or Left Ventricular Dysfunction Insights from the ISCHEMIA Trial. Circulation.

[25] Bangalore S, Maron DJ, Stone GW, Hochman JS (2020). Routine Revascularization Versus Initial Medical Therapy for Stable Ischemic Heart Disease. Circulation.

[26] De Caterina AR, Cuculi F, Banning AP (2013). Incidence, predictors and management of left main coronary artery stent restenosis a comprehensive review in the era of drug-eluting stents. Eur Interv.

[27] Taylor HA, Deumite NJ, Chaitman BR, Davis KB, Killip T, Rogers WJ (1989). Asymptomatic left main coronary artery disease in the Coronary Artery Surgery Study (CASS) registry. Circulation.

[28] Chacón-Diaz M, Vega A, Aráoz O, Ríos P, Baltodano R, Villanueva F (2018). Características epidemiológicas del infarto de miocardio con elevación del segmento ST en Perú resultados del PEruvian Registry of ST-segment Elevation Myocardial Infarction (PERSTEMI). Arch Cardiol Mex.

