# Supplementary material for: Should i treat a high risk chronic coronary syndrome invasively from the beginning? Yes, in most cases
Source: Arch Peru Cardiol Cir Cardiovasc. 2020 Dec 31;1(4):222–8. [Article in Spanish] doi: 10.47487/apcyccv.v1i4.86 (PMC10804820; doi:10.47487/apcyccv.v1i4.86)
Supplement: Related texts

# Reply: Respuesta a Ortiz C. *et al.*

**Authors:** José María Carrasco, René Rodriguez, Luis Murillo, Juan Muñoz, Carlos Alayo

Existen estudios observacionales que describen el beneficio de la estrategia invasiva temprana (EIT) en síndrome coronario crónico (SCC) con isquémica severa, sin embargo muchos tienen cuestionamientos metodológicos ya que incluyen a pacientes con lesión de tronco coronario izquierdo (TCI), grupo que con solo tratamiento médico óptimo conservador (TMOC) tiene elevado riesgo de eventos mayores. Cabe resaltar, que muchos de estos estudios presentan diferencias significativas en las características basales entre los grupos de estudio como edad, sexo, hipertensión arterial, diabetes, dislipidemia, infarto previo y angina así como un mayor uso de aspirina, estatinas, beta bloqueo y antihipertensivos en pacientes con EIT; lo cual podría disminuir la validez interna e inclinar la balanza de eventos mayores hacia el grupo comparativo. Por lo tanto, los resultados del beneficio de la EIT contrastan con sus limitaciones de diseño e inclusión de variables intervinientes; generando incógnitas sobre la influencia de la prevalencia de variables clínicas, uso de fármacos y presencia de lesión severa de TCI del grupo de EIT sobre el impacto clínico en pacientes con SCC e isquemia severa. 

Por otro lado, los ensayos clínicos aleatorizados que comparan EIT versus TMOC (con características basales similares) no evidenciaron diferencias significativas sobre eventos mayores. Así, el COURAGE, con un seguimiento de 15 años, no observó diferencias entre ambos grupos; resultados similares descritos en los estudios MASS y MASS II que también incluyeron pacientes de alto riesgo anatómico como enfermedad coronaria multiarterial quienes representan hasta un 36.4% de pacientes con SCC. Cabe resaltar que estos y otros estudios excluyeron a pacientes con lesión severa de TCI, síndrome coronario agudo reciente, insuficiencia cardiaca con fracción de eyección del ventrículo izquierdo (FEVi) menor al 35% y angina inestable o refractaria a tratamiento médico; indicadores de mal pronóstico cardiovascular (IMPC) cuyo porcentaje en la población de alto riesgo representa menos del 50% y su incidencia es considerablemente baja en pacientes con SCC siendo de 8.7% para lesión de TCI y 10.46% en pacientes con FEVi < 35%, por ello el impacto clínico del TMOC es beneficioso en la mayoría de pacientes de alto riesgo incluyendo ECM y lesión proximal de la descendente anterior.

A estos resultados se añade el estudio ISCHEMIA (que si bien es cierto utilizó dos definiciones de infarto de miocardio (IM), donde hubo mayor riesgo de IM procedimental en la EIT, IM espontáneo en TMOC asociado a mayor riesgo de mortalidad y mayores eventos clínicos en EIT bajo la segunda definición universal de IM), que evidenció que la EIC comparada al TMOC no reduce el riesgo de eventos mayores en pacientes de alto riesgo. Asimismo, los ensayos COURAGE, BARI-2D y FAME-2 no han demostrado reducción del IM espontáneo en TMOC ni en EIT.

Por todo ello, la TMOC inicial debe ser indicada en SCC de alto riesgo sin IMPC (criterios de exclusión en ensayos clínicos previamente mencionados), considerando la posibilidad del manejo invasivo posterior en relación a la presencia de angina refractaria y/o inestable, progresión significativa de la isquemia, evento coronario agudo, disfunción ventricular significativa y otras comorbilidades.
